# Effects of parenting interventions for mothers with depressive symptoms and an infant: systematic review and meta-analysis

**DOI:** 10.1192/bjo.2019.89

**Published:** 2020-01-13

**Authors:** Signe B. Rayce, Ida S. Rasmussen, Mette Skovgaard Væver, Maiken Pontoppidan

**Affiliations:** Senior Researcher, VIVE – The Danish Center for Social Science Research, Denmark; Research Assistant, VIVE – The Danish Center for Social Science Research, Denmark; Associate Professor, Department of Psychology, University of Copenhagen, Denmark; Senior Researcher, VIVE – The Danish Center for Social Science Research, Denmark

**Keywords:** Depressive disorders, child development, parent–child relationship, systematic review, parenting interventions

## Abstract

**Background:**

Postpartum depression is common in the perinatal period and poses a risk for the development of the infant and the mother–infant relationship. Infancy is a critical developmental period of life and supportive parenting is crucial for healthy development, however, the effects of interventions aimed at improving parenting among mothers with depression are uncertain.

**Aims:**

To assess the effects of parenting interventions on parent–child relationship and child development among mothers with depressive symptoms with 0–12-month-old infants.

**Method:**

We conducted a systematic review with the inclusion criteria: (a) randomised controlled trials of structured psychosocial parenting interventions for women with depressive symptoms and a child aged 0–12 months in Western Organisation for Economic Co-operation and Development countries, (b) minimum three sessions with at least half of these delivered postnatally and (c) outcomes relating to the parent–child-relationship and/or child development. Publications were extracted from 10 databases in September 2018 and supplemented with grey search and hand search. We assessed risk of bias, calculated effect sizes and conducted meta-analysis.

**Results:**

Eight papers representing seven trials were included. We conducted meta-analysis on the post-intervention parent–child relationship. The analysis included six studies and showed no significant effect. For individual study outcomes, no significant effects on the majority of both the parent–child relationship and child development outcomes were reported.

**Conclusions:**

No evidence of the effect of parenting interventions for mothers with depressive symptoms was found on the parent–child relationship and child development. Larger studies with follow-up assessments are needed, and future reviews should examine the effects in non-Western countries.

## Maternal depression

Maternal depression is common, and can be experienced during pregnancy (antenatal depression) and after the child is born (postpartum depression). Depression negatively affects the way a person with depression thinks, feels and acts. The symptoms of depression are detected by the use of questionnaires (for example the Edinburgh Postnatal Depression Scale (EPDS)), or diagnosed using a clinical interview such as the DSM or the ICD.^1^ Among mothers in high-income countries, antenatal depression affects 7–12%^2^ and postpartum depression affects 13–18% within the first year after childbirth.^3,4^ Depression causes personal suffering and weakens a person's ability to function in general. A woman with depression in the perinatal period is also faced by the challenges of the antenatal transition into motherhood, and after giving birth, the responsibility of taking care of her infant. Supportive parenting is known to be one of the strongest predictors of good outcomes for children.^5^ Longitudinal studies from many countries show that positive, consistent and supportive parenting predicts low levels of child problem behaviour and child abuse, and also predicts enhanced cognitive development.^6–12^ Conversely, harsh inconsistent parenting predicts a broad range of poor child outcomes.^6,13–16^

Several studies have shown that increased levels of depressive symptoms in the parent are associated with less sensitive and harsher parenting behaviours.^17–19^ Mothers with depression tend to demonstrate a flatter affect and be less sensitive, less responsive and less affectively attuned to their infants' needs, thus violating the infants' basic needs for positive interaction.^20,21^ Maternal depression is a major risk for the infant. The first months of life are a highly sensitive period during which the infant is dependent on maternal care, and during which early brain and socioemotional development take place.^22^ The negative impact of maternal depression on early child development is well documented and includes a broad range of child outcomes and increased susceptibility to psychopathology.^20,23–29^ Maternal depression is frequently considered a unitary construct, but depressive symptoms do not follow a uniform course and there is a great diversity among mothers with depression.^30–32^ In accordance with cumulative risk theories (the more stressors and risk factors a child is exposed to, the bigger their risk of developing mental illness), the most adverse child outcomes are linked to high-risk populations where the depressive symptoms occur in combination with risk factors such as poverty and comorbid psychopathology.^32–35^

The relationships between parental depression and such factors as parental competences, parental sensitivity, parent–child relationship and child development are complex and still not fully explained. Parental depression may influence the child through three potential mechanisms: (a) a direct causal relationship through genetic inheritance of risk genes from parent to child, (b) through shared environmental factors during pregnancy that have an impact on both maternal depression and child development (for example poverty), and (c) through the influence of parental depression on parent behaviour, on the quality of the parent–child relationship and on the overall functioning of the family, which lead to poorer outcomes for the child.^36^

## Existing research

Based on previous studies, we know that interventions that focus solely on the mother (such as medication or psychotherapy targeting the depressive symptoms) are insufficient to buffer against the potentially negative impact of psychopathology on the child's cognitive and psychosocial development, as well as attachment.^37–40^ Previous reviews of the effects of interventions for parents with depressive symptoms that include outcomes on the parent–child relationship or child development outcomes have focused on a broad range of psychological interventions aimed at treating depression in women with antenatal depression or postpartum depression.^39–44^ All reviews explicitly state that their results are either tentative or that evidence is insufficient to draw firm conclusions from. Still, five out of six reviews conclude that the interventions show promising results. Three of the reviews conducted meta-analyses.^41,42,44^

The first review, published in 2011, focused on interventions aimed at enhancing maternal sensitivity, but did not address child developmental outcomes.^41^ The second review, published in 2015, focused on psychological treatment of depression in mothers.^42^ The study included meta-analyses of both the mother–child relationship and child mental health outcomes, but mixed observational and parent-reported measures in the analyses. The third review, published in 2017 by Letourneau and colleagues, focused on interventions aimed at treating perinatal depression.^44^ Their meta-analysis focused only on the effect of two types of interventions: interpersonal psychotherapy and cognitive–behavioural therapy (CBT). Each meta-analysis included two studies only, which is not ideal.^45^

We found no previous reviews focusing specifically on the effect of interventions aimed at improving parenting including both the parent–child relationship and child development outcomes. The objective of this review is therefore to systematically review the effects of parenting interventions on the parent–child relationship and child development outcomes when offered to pregnant women or mothers with depressive symptoms who have infants aged 0–12 months. We included randomised controlled trials (RCTs) of interventions that aimed at improving parenting in a broad sense (such as Circle of Security^46^ or Minding the Baby^47^) and that reported on the parent–child relationship (for example attachment or parent–child relationship) or child development (for example socioemotional or cognitive development) outcomes at post-intervention or follow-up.

## Method

This review was conducted according to the Preferred Reporting Items for Systematic Reviews and Meta-Analyses (PRISMA). We did not register a protocol.

### Search strategy

The latest database search was performed in September 2018. Ten international bibliographic databases were searched: Campbell Library, Cochrane Library, CRD (Centre for Reviews and Dissemination), ERIC, PsycINFO, PubMed, Science Citation Index Expanded, Social Care Online, Social Science Citation Index and SocIndex. Operational definitions were determined for each database separately. The search strategy was developed in collaboration between an information specialist and a member of the review team (M.P.), and comprised three separate reviews on parenting interventions.^48,49^ The main search comprised combinations of the following terms: infant*, neonat*, parent*, mother*, father*, child*, relation*, attach*, behavi*, psychotherap*, therap*, intervention*, train*, interaction, parenting, learning and education (see supplementary File 1 available at https://doi.org/10.1192/bjo.2019.89). To identify studies on women with depression or depressive symptoms in the perinatal period, the search also included the term depress*. The searches included Medical Subject Headings (MeSH), Boolean operators and filters. Publication year was not a restriction. Furthermore, we searched for grey literature, hand searched four journals and snowballed for relevant references.

### Eligibility criteria and study selection

All publications were screened based on title and abstract. Publications that could not be excluded were screened based on the full-text version. Each publication was screened independently by two research assistants under close supervision by S.B.R. and M.P. Uncertainties regarding inclusion were discussed with S.B.R. or/and M.P. Screening was performed in Eppi-Reviewer 4. The inclusion and exclusion criteria are presented in Table 1.

**Table 1:** Inclusion and exclusion criteria

Inclusion criteria	Exclusion criteria
Population	
Depression or depressive symptoms in mothers of infants 0–12 months old in Western Organisation for Economic Co-operation and Development countries.	Studies including young mothers (mean age <20 years), parents with severe mental health problems such as schizophrenia, parents with children born preterm, at low birth weight or with congenital diseases, or studies that included mothers without depressive symptoms.
Intervention	
Structured psychosocial parenting intervention consisting of at least three sessions and initiated either antenatal or during the child's first year of life with at least half of the sessions delivered postnatally.	Interventions not focusing specifically on parenting (for example baby massage, cognitive–behavioural therapy, or breastfeeding interventions), and unstructured interventions (for example home visits not offered in a structured format).
Control group	
No restrictions were imposed. All services or comparison interventions provided to the control group were allowed.	
Outcome	
Child development and/or parent–child relationship outcomes.	Studies reporting only physical development or health outcomes such as height, weight, duration of breastfeeding and admissions to hospital. Papers with insufficient quantitative outcome data to generate standardised mean differences (Cohen's *d*), risk ratios and confidence intervals.
Design	
Randomised controlled trials (RCT) or quasi-RCTs.	Other study designs such as case–control, cohort, cross-sectional and systematic reviews.
Publication type	
Studies presented in peer-reviewed journals, dissertations, books or scientific reports.	Abstracts or conference papers. Studies published in languages others than English, German or the Scandinavian languages (Danish, Swedish and Norwegian).

### Data extraction and risk of bias assessment

We developed a data extraction tool for the descriptive coding and extracted information on (a) study design, (b) depression inclusion criteria, (c) sample characteristics, (d) intervention characteristics, (e) setting, (f) outcome measures, and (g) child age at post-intervention and at follow-up. The information was extracted by a research assistant and checked by S.B.R. Primary outcomes were (a) parent–child relationship and (b) child socioemotional development. Secondary outcomes comprised other child development markers, for example cognitive and language development. The numeric coding was conducted independently by two reviewers (S.B.R. and I.S.R.). Disagreements were resolved by discussion, and, if necessary, a third reviewer was consulted.

We assessed risk of bias separately for each relevant outcome for all studies based on a risk of bias model developed by Professor Barnaby Reeves and the Cochrane Non-randomised Studies Method Group (Reeves, personal communication, 2019 from Reeves, Deeks, Higgins and Wells, unpublished data, 2011). This extended model follows the same steps as the risk of bias model presented in the Cochrane Handbook, Chapter 8.^50^ The risk of bias assessment was conducted by I.S.R. and checked by S.B.R. Any doubts were resolved by consulting a third reviewer.

### Data analysis

We calculated effect sizes for all relevant outcomes where sufficient data was provided. Effect sizes are reported using standardised mean differences (Cohen's *d*) with 95% CI for continuous outcomes. Data include post-intervention and follow-up means (or mean differences), raw s.d.s and sample size. For dichotomous outcomes, we used risk ratios (RR) with 95% CI. If a paper provided insufficient information regarding numeric outcome, the corresponding author was contacted. When available, we used data from adjusted analyses to calculate effect sizes. When adjusted mean differences were calculated, we used the unadjusted s.d.s to be able to compare effect sizes calculated from unadjusted and adjusted means. To calculate effect sizes, we used the Practical Meta-Analysis Effect Size Calculator developed by David B. Wilson, George Mason University and provided by the Campbell Collaboration.^51^

Meta-analysis was conducted when outcome and time of assessment were comparable. When a single study provided more than one relevant measure, or only subscales of an overall scale for the meta-analysis, the effect sizes of the respective measures were pooled into a joint measure before being entered in the meta-analysis. One study consisted of three separate intervention arms and a shared control group.^52^ Since three effect sizes based on the same control group would not be independent, we calculated an effect size based on a mean of the three intervention groups' means and the mean of the control group.

Random-effects inverse-variance-weighted mean effect sizes were applied and 95% CIs were reported. Thus, studies with larger sample sizes were given more weight, all else being equal. Based on the relatively small number of studies and on an assumption of between-study heterogeneity, we used a random-effects model using the profile-likelihood estimator as suggested in Cornell.^53^ Variation in standardised mean difference that was attributable to heterogeneity was assessed with the *I*^2^. The estimated variance of the true effect sizes was assessed by the Tau^2^ statistic. The small number of studies in the meta-analysis did not allow for subgroup analyses.

Assessment times were divided into post-intervention (at intervention ending), short-term (less than 12 months after intervention ending) and long-term (12 months or more) follow-up.

## Results

**Fig. 1:**
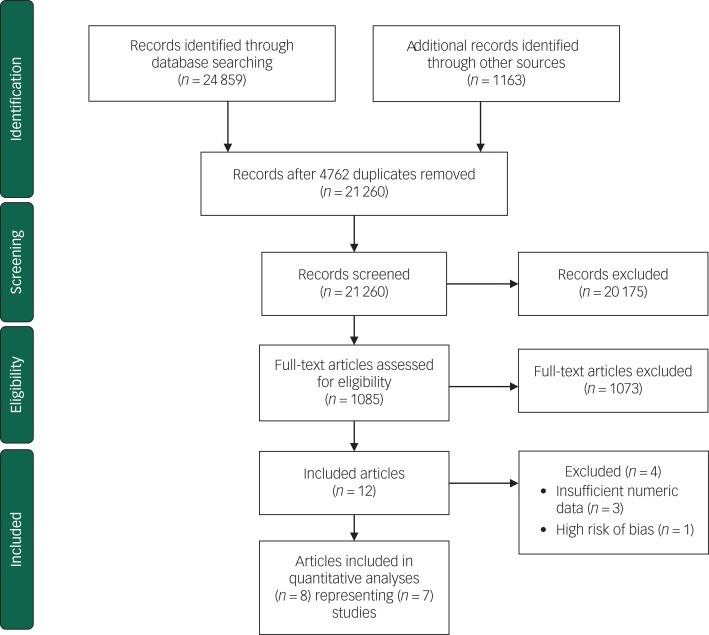
Flow diagram for study selection process.^54^

The search identified 21 260 articles after removal of duplicates. After first- and second-level screening 12 articles remained. A further three articles were excluded because of insufficient numerical data and one was excluded because of high risk of bias. See Fig. 1 for a flow chart of the process. Seven randomised controlled trials (eight published papers) met all inclusion criteria and were included in this review.^52,55–61^

All studies were randomised at the individual level. Three studies were American,^55–57^ two were British,^52,61^ one was Canadian^58^ and one study presented in two papers was Dutch.^59,60^ Three studies were excluded because of insufficient numeric data^62–64^ and one study was excluded because of unacceptably high risk of bias.^65^

### Participant characteristics

Table 2 presents participant characteristics. No studies started during pregnancy. All women were included based on specific inclusion criteria for level of depressive symptoms. Four studies used the EPDS,^55–58^ one study used the EPDS or a DSM-II-R major depressive disorder diagnosis,^52^ one study used a diagnosis of major depressive disorder,^61^ and one study used the Beck Depression Inventory or DSM-IV major depressive episode or dysthymia diagnosis^59,60^ as inclusion criteria. The mean age of the mothers at inclusion ranged from 27.7 to 32 years and mean age of the infant between 1 and 7 months. Two studies included primiparous mothers only,^52,55^ whereas the other five included both primiparous and multiparous mothers.^56,57,59–61,66^ In most studies, the majority of participants were White, married/living with partner, and with a medium or long education. All studies were relatively small, ranging from 42 to 190 participants.

**Table 2:** Participant characteristics

Study	Country	Child age at start	Mother, mean age	Depression inclusion criteria	Primiparous, %	Socioeconomic status	*n*
Goodman *et al*^55^	USA	6–8 weeks	30.7	>9 and <20 on the EPDS scale	100	White 60%, Hispanic 24%; 87% with income >$40 000; education: low 7%, medium 66%, high 27%; desired pregnancy 90%	42
Horowitz *et al*^56^	USA	4–8 weeks	31	EPDS >10	66	Community sample Boston, USA; well educated; majority White (69%); 71% with income >$50 000	117
Horowitz *et al*^57^	USA	6 weeks	31	EPDS ≥10	56	Community sample Boston, USA; White 54%, Hispanic 22%, African American 12%, other 13%; married 75%; mean income $80 132; well educated (mean: 15.6 years (s.d. = 3.4))	134
Letourneau *et al*^58^	Canada	Mean 5.2 months	~30	EPDS >12	47	Living near Alberta or New Brunswick, Canada; College degree or more 65%; married 67%; 55% with income <$40 000	60
Murray *et al*^52^	UK	8 weeks postpartum	27.7	EPDS >12 and DSM-III-R: major depressive disorder diagnosis	100	Community sample; living <15 miles from Addenbrooke's Hospital, Cambridge, UK; education: low 45%, middle 31%, high 24%; high social disadvantage 25%	190
Stein *et al*^61^	UK	Mean 6.8 months	32	Major depressive disorder	45	Living in Oxfordshire, Buckinghamshire and Berkshire counties, UK; White British 83%, other 17%; education: low 41%, medium 11%, high 48%; living with father 88%	144
Van Doesum *et al*,^59^ Kersten-Alvarez *et al*^60^	The Netherlands	Mean 5.5 months	30.0	DSM-IV: major depressive episode or dysthymia and/or BDI >14	60	Majority Dutch; education: low 25%, middle 50%, high, 25%; income: low 21%, middle 55%, high 24%; living with partner 90%	71

EPDS, Edinburgh Postnatal Depression Scale; BDI, Beck Depression Inventory.

### Intervention characteristics

Table 3 presents intervention characteristics, assessment times and outcomes. All included studies offered individual home visits initiated postpartum. No studies offered a group-based intervention. Most interventions focused on supporting the parent–child relationship through for example video feedback, coaching or therapy. Four studies offered alternative treatment such as progressive muscle relaxation, home visits or phone calls to the control group.^55,57,59,61^ One study also provided CBT for both the intervention and control group.^61^ Control condition was usual care in two studies^52,56^ and waiting list in one study where participants received usual care while waiting.^58^ Besides one intervention lasting 7.5 months,^56^ all interventions were relatively short (3–15 weeks) and the intensity ranged between weekly visits to one visit a month.

**Table 3:** Intervention characteristics, assessment times and outcomes

Study	Intervention	Child age at start	Format	Intensity and duration	Provider	Control condition	Child age at assessment (months)	Outcome categories
Goodman *et al*^55^	Perinatal dyadic psychotherapy	6–8 weeks	Home visits	8 visits over 3 months	Nurse	Usual care and phone calls	4.5–5 (post-intervention) 7.5–8 (short term)	Parent–child relationship
Horowitz *et al*^56^	Interaction coaching intervention	4–8 weeks	Home visits	3 visits over 15 weeks	Nurse	Usual care	3–4 (post-intervention)	Parent–child relationship
Horowitz *et al*^57^	Communicating and relating effectively	6 weeks	Home visits	6 visits over 7.5 months	Nurse	4 home visits	9 (post-intervention)	Parent–child relationship
Letourneau *et al*^58^	Peer-support intervention	<9 months	Home visits and telephone contacts	12 visits over 12 weeks	Trained peer volunteers	Wait-list and usual care	~8 (post-intervention)	Child development Parent–child relationship
Murray *et al*^52^	Cognitive–behavioural therapy, psychodynamic therapy and non-directive counselling	8 weeks	Home visits	Weekly visits for 10 weeks	Therapist	Usual care by GP and health visitor	4.5 (post-intervention) 18 (long term) 60 (long term)	Child development Parent–child relationship
Stein *et al*^61^	Video-feedback therapy and cognitive–behaviour therapy	4.5–9 months	Home visits or alternative location	11 visits over 16 weeks, 2 booster sessions at 6 and 10 months	Therapist	Progressive muscle relaxation and cognitive–behaviour therapy	~12 (post-intervention) ~24 (long term)	Child development Parent–child relationship
Van Doesum *et al*,^59^ Kersten-Alvarez *et al*^60^	Video-feedback intervention	<12 months	Home visits	8–10 visits over 3–4 months	Prevention specialist	3 phone calls over 3 months	~12 (post-intervention) ~18 (short term) 68 (long term)	Child development Parent–child relationship

Goodman and colleagues (2015) examined the effects of Perinatal Dyadic Psychotherapy among 42 mothers recruited from postpartum units of three hospitals located in the USA.^55^ The study was a pilot study to examine a novel dual-focused mother–infant intervention aimed at promoting maternal mental health and improving the relationship between mother and infant. The intervention was derived from the mutual regulation model by Tronick,^67^ and integrates clinical strategies of supportive psychotherapy, parent–infant psychotherapy, the touchpoints model of child development^68^ and the newborn behavioral observation.^55,69^

Horowitz and colleagues (2001) examined the effects of an interactive coaching intervention among 117 mothers from greater Boston in the USA.^56^ The aim of the intervention was to improve responsiveness between infant and mother. The intervention is based on Beck's cognitive model of depression,^70^ Sameroff's transactional model of child development^71^ and Rutter's model of developmental risk and resilience.^56^

Horowitz and colleagues (2013) examined the effects of the behavioural coaching intervention communicating and relating effectively on 134 mother–infant dyads in the USA.^57^ The intervention aimed to improve the mother–infant relationship. The intervention is based on cognitive–behavioural family therapy theory.^57^

Letourneau and colleagues (2011) examined the effects of home-based peer support among 60 mothers in Canada.^58^ The home-based peer support included mother–infant interaction teaching as an important element and the intervention aimed to improve interactions between mothers and their infants. The intervention is not based on any specific theory, but the authors refer to concepts from studies on mothers with postpartum depression and peer support.^58^

Murray and colleagues (2003) examined the effects of three different, but related, interventions: (a) CBT, (b) psychodynamic therapy and (c) non-directive counselling in one trial in the UK.^52^ In total, 190 first-time mothers were included and almost equally distributed among the three intervention groups and the single control group. The interventions all aimed to improve the mother–child relationship. The CBT intervention was a modified form of interaction guidance treatment. In the psychodynamic therapy intervention, the mother's representations were used to explore her own attachment history. The non-directive counselling intervention provided the mothers with an opportunity to talk about their feelings about current concerns.^52^

Stein and colleagues (2018) examined the effects of video-feedback therapy among 144 mothers in the UK. The aim of the intervention was to improve parenting behaviours (attention to infant cues, emotional scaffolding and sensitivity). Both intervention and control families also received CBT with a focus on behavioural activation. The control group received progressive muscle relaxation that did not target parent practices or parent–child interaction.^61^

Van Doesum and colleagues (2008) examined the effects of a mother–baby intervention among 71 mothers in the Netherlands.^59,60^ The intervention aimed to improve the mother–infant interaction, particularly maternal sensitivity. The intervention was based on video feedback, modelling, cognitive restructuring, practical pedagogical support and baby massage.^59,60^

### Outcome characteristics

All seven studies examined parent–child relationship outcomes. Measures applied to assess parent–child relationship (for example attachment or parent–child relationship) included the Emotional Availability Scales (EAS),^59^ Coding Interactive Behavior (CIB),^55^ the Nursing Child Assessment Teaching Scale^57^ and the Dyadic Mutuality Code.^56^ Four studies assessed child development outcomes (such as socioemotional or cognitive development) with measures such as the Infant-Toddler Social Emotional Assessment (ITSEA),^59^ the Child Behavior Checklist (CBCL)^60,61^ and Bayley Scales of Infant Development (BSID).^61^ Outcomes were assessed by independent assessors (for example EAS, CIB, and BSID), by parents (for example ITSEA and CBCL), and/or teachers (for example CBCL). All seven studies included a post-intervention assessment, but only four studies included a follow-up assessment.^52,55,59–61^ As the development of maternal depressive symptoms is not the focus of this review, we did not include maternal depression in our analyses, although it was reported in all studies.

### Risk of bias

Risk of bias assessments for each study's outcomes are displayed in online supplementary Table 1 divided into parent–child relationship and child development outcomes. Six out of seven studies provided insufficient information for one or more risk of bias domain, thus hindering a clear risk of bias judgement. Only two studies^60,61^ provided an accessible *a priori* protocol, which made it particularly difficult to assess risk of bias caused by selective reporting. In general, risk of bias ranged between low and medium. Five studies had outcomes where one or two domains were classified as medium risk of bias.^52,55,57,58,61^ Only one study had outcomes with a high risk of bias in one domain: ‘incomplete outcome data addressed’.^60^ One study was excluded from the review because of an unacceptably high risk of bias caused by lack of assessor masking and high risk of bias in relation to ‘incomplete outcome data addressed’.^65^

The outcomes included in the meta-analysis on the parent–child relationship were characterised by low-to-medium and unclear risk of bias domain. In three of these studies,^52,56,59^ the risk of bias domains of the outcomes were assessed as relatively low (1–2) or unclear risk of bias. The outcomes of the remaining three studies^55,57,58^ were characterized by low-to-medium and unclear risk of bias.

### Parent–child relationship

Seven studies reported on parent–child relationship outcomes.^52,55–59,61^ supplementary Table 2 presents the study outcomes for the individual studies. Owing to the lack of follow-up assessments, only meta-analysis of the parent–child relationship post-intervention was conducted.

### Post-intervention parent–child relationship

**Fig. 2:**
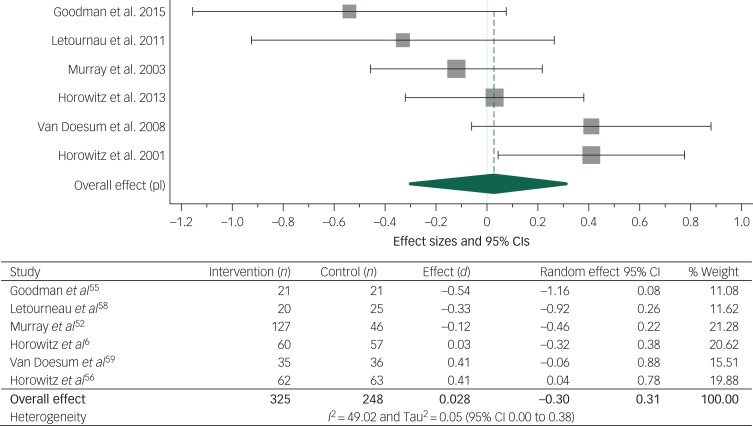
Meta-analysis of studies reporting parent–child relationship at post-intervention.

The meta-analysis of the effect of the parenting intervention on the parent–child relationship at post-intervention included 573 participants from six studies and is presented in Fig. 2.^52,55–59^ No significant effect of the parenting interventions on the parent–child relationship was found (*d* = 0.028, 95% CI −0.30 to 0.31) (*I*^2^ = 49.02). One study^58^ that provided a peer-support intervention instead of using professional providers was removed for sensitivity analysis. This did not alter the result substantially. Likewise, three studies offering alternative treatment for the control group^55,57,59^ were removed for sensitivity analysis. The result of the meta-analysis of the three remaining studies that provided treatment as usual^52,56,58^ was not altered substantially. All six studies were therefore kept in the analysis.

One out of the two parent–child relationship outcomes in the study by Murray *et al*^52^ was based on a parent-reported questionnaire. As all other outcomes included in the meta-analysis were observational measures, this outcome was not included in the analysis. When examining relationship problems, Murray *et al* (2003) found significant positive effects of counselling, psychodynamic therapy and CBT compared with usual care (counselling: RR = 0.63, 95% CI 0.32–0.97); psychodynamic: RR = 0.57, 95% CI 0.28–0.92); cognitive–behavioural: RR = 0.46, 95% CI 0.20–0.81).^52^

### Parent–child relationship at short-term follow-up

Two studies reported on the parent–child relationship at short-term follow-up.^55,59^ Van Doesum and colleagues^59^ found significant effects of the mother–infant intervention on maternal sensitivity (*d* = 0.82, 95% CI 0.34–1.31), maternal structuring (*d* = 0.57, 95% CI 0.09–1.04), child responsiveness (*d* = 0.69, 95% CI 0.21–1.16) and child involvement (*d* = 0.75, 95% CI 0.27–1.23) at short-term follow-up when the children were around 18 months old. No significant effects were found on child attachment security, maternal non-intrusiveness or maternal non-hostility.^59^ Goodman and colleagues likewise measured maternal sensitivity and infant involvement but found no significant effects on maternal sensitivity, infant involvement or dyadic reciprocity when the children were around 8 months old.^55^

### Parent–child relationship at long-term follow-up

Three studies reported on the parent–child relationship at long-term follow-up.^52,60,61^ Meta-analysis was not conducted, as the study by Stein and colleagues used an active control group, whereas the control groups in the two remaining studies received either alternative treatment or treatment as usual. Kersten-Alvarez and colleagues found no effects on maternal interactive behaviour or attachment security when the children were 4 years old.^60^ Murray and colleagues found that none of the three interventions provided had a significant effect on child attachment at long-term follow-up (child aged 18 months) when compared with the control group.^52^ Stein and colleagues found no significant effects on attachment security for children aged 2 years.^61^

### Child development

Four studies reported on child development outcomes. Supplementary Table 3 presents the study outcomes for the individual studies. Owing to the lack of developmental outcomes and follow-up assessments, it was not possible to conduct meta-analysis of child development.

### Post-intervention child development

Two studies examined child development post-intervention.^52,58^ Letourneau and colleagues found no significant effect on child cognitive and socioemotional development.^58^ Murray and colleagues found that none of the three interventions provided had a significant effect on infant behaviour problems post-intervention.^52^

### Child development at short-term follow-up

One study, van Doesum and colleagues, examined child development at short-term follow-up.^59^ They found a significant effect of home visits on child competence behaviour (*d* = 0.62, 95% CI 0.14–1.10), but no significant effects on externalising, internalising or dysregulated behaviour.^60^

### Child development at long-term follow-up

Three studies examined child development at long-term follow-up (range: 13–56 months).^52,60,61^ Murray and colleagues^52^ found significant positive effects of the counselling intervention (*d* = 0.64, 95% CI 0.22–1.05) and the psychodynamic intervention (*d* = 0.49, 95% CI 0.07–0.91) on emotional and behavioural problems when the children were 18 months old, but no effects of the cognitive–behavioural intervention at 18 months old. When the children were 60 months old, a positive significant effect of the cognitive–behavioural intervention on mother-rated emotional and behavioural difficulties (*d* = 0.61, 95% CI 0.12–1.11) was found, but not on teacher-rated emotional and behavioural difficulties. No significant effects of the two other interventions were found. None of the three interventions showed significant effects on child cognitive development at any of the two follow-up assessments. Kersten-Alvares and colleagues found no significant effects of the intervention on child self-esteem, verbal intelligence, prosocial behaviour, school adjustment and behaviour problems at long-term follow-up.^60^ Stein and colleagues found no significant effects on cognitive and language development, behaviour problems, attention focusing, attentional shifting, inhibitory control and child emotion regulation.^61^

## Discussion

### Main findings

We identified eight papers representing seven trials that examined the effects of parenting interventions offered to pregnant women or mothers with depressive symptoms. As a result of the variety of assessment measures and study designs, only meta-analysis on the parent–child relationship at post-intervention was performed. We found no significant effect of parenting interventions on the parent–child relationship. When we examine the individual study outcomes, there was no significant effect on most of the child development and parent–child relationship outcomes reported in the papers.

Only two studies found significant effects on a child development outcome, both positive.^52,59^ Four studies found significant effects on a parent–child relationship outcome, three positive and one negative, ranging between a large negative effect on infant involvement and a large positive effect on child responsiveness.^52,55,56,59^ The general lack of effect of the interventions aimed at mothers with depressive symptoms is consistent with previous reviews that examine a number of different types of interventions for mothers.^39–44^ Consequently, there is a need to consider whether the interventions currently offered to mothers with depressive symptoms are appropriate, or if other strategies should be tried out.

Previous research shows that the most adverse child outcomes are found in high-risk populations where the depressive symptoms occur in combination with poverty and comorbid psychopathology.^32–35^ Families with a relatively high socioeconomic status may therefore not be affected as severely by postpartum depression as families with low socioeconomic status. The socioeconomic status of the families included in the studies of this review is relatively high; most participants are of White ethnicity, and only a relatively small number of the participants have no education or limited education and/or a low income. This may contribute to the non-significant effect on the parent–child relationship found in the meta-analysis and the generally non-significant results of the individual studies.

### Interpretation of our findings and avenues for further research

Most studies included in this review used a screening questionnaire such as the EPDS to measure the level of depressive symptoms. A questionnaire is easy to use, but is also less precise than a depression diagnosis based on a clinical interview. Although the participants included in this review had a relatively high score on a depression screening questionnaire, they do not necessarily fulfil criteria for a clinical depression. The interventions might be effective if they were examined with a group of mothers with clinical depression, as such mothers may profit more from the intervention. Future studies could therefore be aimed at specific high-risk populations such as socioeconomically deprived mothers or women with clinical depression to examine if interventions are effective within such populations. Although there is a high correlation between antenatal depression and postpartum depression,^72^ and the relationship with the child starts to form during pregnancy, none of the interventions of the included studies were initiated during pregnancy.^73^ Therefore, screening for depression in pregnancy and starting treatment before the baby is born could be a focus for future research.

Previous reviews have pointed out that existing studies on psychological interventions for pregnant women or mothers with depression are few and have small sample sizes.^40,41,43^ Sample sizes of the studies included in this review ranged from 42 to 190 participants, which may have limited the power to detect significant effects. Although four of the studies included in this review include 100–190 participants, the studies are generally still small in sample size and limited in number in this updated review. We did not find any systematic differences according to study size. Future studies should have a larger sample size to increase power. In order to examine mechanisms of change, careful considerations about the need for moderator or mediator analyses should be made *a priori*, as this increases the required sample size considerably.^74,75^ Examples of possible moderators are socioeconomic status and depression level, and a possible mediator could be reflective functioning or sensitivity, depending on the theory of change in the examined intervention.

Several factors may moderate the effect of parenting interventions. First, intervention characteristics such as theoretical background may moderate the effect of parenting interventions. The interventions included in this review were, however, relatively comparable with regard to delivery, theoretical background and intensity. Most were home visits conducted by trained therapists, with weekly to monthly visits for a relatively short period.

Second, group format is widely used in parenting interventions and achieves change through the dual process of emotional experience and reflection in an interpersonal context.^76,77^ Group sessions provide a support network, reduce isolation and stigma, provide an environment in which to practice interpersonal and communication skills, and shape coping strategies and learning from each other. This may be important for mothers with depression as they may feel alone with their problems. We did not, however, find any studies that employed a group format. A group intervention (circle of security – parenting) offered to mothers with depression is currently being evaluated in a RCT in Denmark.^78^ The group format enables several families to be treated at once, making it cheaper than individual interventions.

Finally, in recent years both practice and research have become much more aware of the important role fathers can play.^79^ The father–child relationship might be especially important to both the mothers and the children in the context of maternal problems, such as depression, in which the father might buffer negative effects on children's socioemotional development. At the same time, non-optimal paternal behaviour and father–child relationships might act as an additional risk factor for problematic child development.^31,80,81^ It is therefore crucial to consider how fathers can be involved in the support offered to new families. None of the studies in this review, however, included fathers in the intervention.

### Limitations

We chose to include only RCTs or quasi-RCTs in the review to ensure high methodological quality and to minimise the risk of confounding factors. We consider this a strength of the review, but it may have reduced the number of included studies, thereby making it more difficult to find comparable studies for meta-analyses. Likewise, the small number of included studies hindered subgroup analysis, which must be considered a limitation. Although some studies did report short- or long-term follow-up outcomes, it was not possible to conduct meta-analysis on any follow-up outcomes. Consequently, we cannot say anything about the effects over time.

Another limitation that stems from the reviewed studies is that although the two most recent studies^55,61^ addressed implementation issues such as details about certification, supervision, fidelity and variation in the number of intervention sessions received, most studies only provide limited information about training. Therefore, when comparing across studies, we do not have a clear picture of how well the interventions were delivered and whether the results could have been affected by implementation difficulties. A final limitation is that only studies conducted in Western Organisation for Economic Co-operation and Development countries were included in this review. Since cultural norms and values related to parenting vary considerably across countries,^82^ we chose to focus on the effectiveness of interventions offered to families in high-income countries in this review. However, the majority of the world's population lives in low- and middle-income countries. Therefore, it is important to conduct similar reviews focusing on studies from low- and middle-income countries.

### Implications for practice

This review, based on seven studies, provides no evidence for the effect of parenting interventions for mothers with depressive symptoms on the parent–child relationship immediately after the intervention ended. As meta-analysis for child development or follow-up assessments could not be done, it remains unclear whether there are any effects on these outcomes. Despite the current accepted need to intervene within the first 1000 days of a vulnerable child's life, and the fact that parental depression can have serious developmental consequences for the child, we still lack high-quality studies to inform practice about how best to support vulnerable families. We also still lack systematic reviews that examine the effects of interventions for mothers with depression outside high-income countries.

## Supplementary material

For supplementary material accompanying this paper visit http://dx.doi.org/10.1192/bjo.2019.89. click here to view supplementary material
