# Supplementary material for: Effects of parenting interventions for mothers with depressive symptoms and an infant: systematic review and meta-analysis
Source: BJPsych Open. 2020 Jan 13;6(1):e9. doi: 10.1192/bjo.2019.89 (PMC7001473; doi:10.1192/bjo.2019.89)
Supplement: Supplementary file 1 [file S2056472419000899sup001.zip › BJO_1900089_supplementaryTables2and3.docx]

**Table 2** Parent–child relationship outcomes as reported across studies in the systematic review

| Study | Measure | Assessment | Child age in months | Intervention | | | Control | | | Effect size | | Other statistics |
| --- | --- | --- | --- | --- | --- | --- | --- | --- | --- | --- | --- | --- |
|  |  |  |  | *N* | Mean | s.d. | *N* | Mean | s.d. | Cohen’s d (95% CI) | RR (95% CI) |  |
| Post-intervention | | | | | | | | | | | | |
|  | | | | | | | | | | | | |
|  | | | | | | | | | | | | |
| Horowitz 2001 | DMC Responsiveness, mother–infant | V | 3–4 | 60 | 9.55 | 1.77 | 57 | 8.80 | 1.86 | **0.41 (0.05;0.78)** |  |  |
| Horowitz 2013^a^ | NCATS Relational effectiveness, mother–infant | V | 9 | 62 | 53.16 | 8.3 | 63 | 53.71 | 7.0 | −0.07 (−0.42;0.28) |  |  |
|  | NCATS Child responsiveness | V | 9 | 62 | 7.44 | 3.1 | 63 | 7.03 | 3.1 | 0.13 (−0.69;0.95) |  |  |
| Letournau 2011^a^ | NCAST PCI feeding scale Mother–infant interaction | V | ~8 | 19 | 57.5 | 8.26 | 24 | 59.0 | 7.90 | −0.19 (−0.79;0.42) |  |  |
|  | NCAST PCI teaching scale Mother–infant interaction | V | ~8 | 21 | 50.4 | 8.13 | 25 | 53.6 | 5.76 | −0.46 (−1.05;0.13) |  |  |
| Van Doesum 2008^a^ | EAS Maternal sensitivity | V | ~12 | 35 | 4.82 | 1.78 | 36 | 3.79 | 1.86 | **0.57 (0.09; 1.04)** |  |  |
|  | EAS Maternal structuring | V | ~12 | 35 | 3.12 | 1.09 | 36 | 2.71 | 1.06 | 0.38 (−0.09; 0.85) |  |  |
|  | EAS Maternal non-intrusiveness | V | ~12 | 35 | 3.56 | 1.56 | 36 | 3.24 | 1.15 | 0.23 (−0.23; 0.70) |  |  |
|  | EAS Maternal non-hostility | V | ~12 | 35 | 4.89 | 0.41 | 36 | 4.84 | 0.56 | 0.10 (−0.36; 0.57) |  |  |
|  | EAS Child responsiveness | V | ~12 | 35 | 4.26 | 1.48 | 36 | 3.18 | 1.74 | **0.67 (0.19; 1.15)** |  |  |
|  | EAS Child involvement | V | ~12 | 35 | 3.74 | 1.83 | 36 | 2.79 | 1.91 | **0.51 (0.04; 0.98)** |  |  |
| Murray 2003^b^ | Global rating scale Murray Mother–infant relationship | V | 4.5 |  |  |  |  |  |  |  |  |  |
| Counselling | Relationship problems (in-house) |  |  | 46 | 3.02 | 0.58 | 46 | 2.96 | 0.75 | 0.09 (−0.32; 0.50) |  |  |
| Psyhodynamic |  |  |  | 43 | 2.80 | 0.68 | 46 | 2.96 | 0.75 | −0.22 (−0.64; 0.19) |  |  |
| Cognitive behavioural |  |  |  | 38 | 2.82 | 0.75 | 46 | 2.96 | 0.75 | −0.19 (−0.62; 0.24) |  |  |
|  |  | Q | 4.5 |  |  |  |  |  |  |  |  |  |
| Counselling |  |  |  | 40 |  |  | 31 |  |  |  | **0.63 (0.32–0.97)**^c^ |  |
| Psyhodynamic |  |  |  | 40 |  |  | 31 |  |  |  | **0.57 (0.28–0.92)^c^** |  |
| Cognitive behavioural |  |  |  | 41 |  |  | 31 |  |  |  | **0.46 (0.20–0.81)^c^** |  |
| Goodman 2015^54a^ | CIB Maternal sensitivity | V | ~18–20 | 21 | 3.69 | 0.59 | 21 | 3.95 | 0.55 | −0.46 (−1.07; 0.16) |  |  |
|  | CIB Infant involvement | V | ~18–20 | 21 | 3.20 | 0.69 | 21 | 3.79 | 0.52 | −**0.97(−1.60; −0.33)** |  |  |
|  | CIB Dydic reciprocity | V | ~18–20 | 21 | 3.46 | 0.68 | 21 | 3.60 | 0.83 | −0.18 (−0.79; 0.42) |  |  |
| Short term | | | | | | | | | | | | |
| Van Doesum | AQS attachment |  | ~18 | 35 | 0.41 | 0.30 | 36 | 0.26 | 0.35 | 0.46 (−0.01; 0.93) |  |  |
|  | EAS Maternal sensitivity | V | ~18 | 35 | 5.18 | 2.01 | 36 | 3.63 | 1.76 | **0.82 (0.34; 1.31)** |  |  |
|  | EAS Maternal structuring | V | ~18 | 35 | 3.12 | 1.09 | 36 | 2.44 | 1.30 | **0.57 (0.09; 1.04)** |  |  |
|  | EAS Maternal non-intrusiveness | V | ~18 | 35 | 3.57 | 1.27 | 36 | 3.11 | 1.40 | 0.34 (−0.12; 0.81) |  |  |
|  | EAS Maternal non-hostility | V | ~18 | 35 | 4.80 | 0.67 | 36 | 4.81 | 0.52 | −0.02 (−0.48; 0.45) |  |  |
|  | EAS Child responsiveness | V | ~18 | 35 | 4.60 | 1.77 | 36 | 3.52 | 1.36 | **0.69 (0.21; 1.16)** |  |  |
|  | EAS Child involvement | V | ~18 | 35 | 4.57 | 1.91 | 36 | 3.25 | 1.59 | **0.75 (0.27; 1.23)** |  |  |
| Goodman 2015 | CIB Maternal sensitivity | V | ~30–32 | 21 | 3.73 | 0.84 | 21 | 3.88 | 0.66 | −0.20(−0.80; 0.41) |  |  |
|  | CIB Infant involvement | V | ~30–32 | 21 | 3.34 | 0.78 | 21 | 3.66 | 0.48 | −0.49(−1.11; 0.12) |  |  |
|  | CIB Dyadic reciprocity | V | ~30–32 | 21 | 3.72 | 0.97 | 21 | 3.73 | 0.91 | −0.01(−0.62; 0.59) |  |  |
| Long term | | | | | | | | | | | | |
| Kersten-Alvarez 2010 | Erickson and Smeeken Maternal interactive behaviour | V | ~68 | 29 |  |  | 29 |  |  | −0.34 (−0.86; 0.17) |  | Dif score: −0.48, s.d. of DV: 1.43^d^ |
|  | ASCT attachment | V | ~68 | 29 |  |  | 29 |  |  | 0.08 (−0.43; 0.60) |  | Dif score: 0.31, s.d. of DV: 3.88^d^ |
| Murray 2003 | ASSP attachment | V | 18 |  |  |  |  |  |  |  |  |  |
| Counselling |  |  |  | 39 |  |  | 47 |  |  |  | 0.96 (0.54–1.46) |  |
| Psyhodynamic |  |  |  | 40 |  |  | 47 |  |  |  | 1.23 (0.76–1.68) |  |
| Cognitive behavioural |  |  |  | 41 |  |  | 47 |  |  |  | 1.26 (0.78–1.70) |  |
| Stein 2018 | AQS Attachment | V | ~24 | 64 |  |  | 68 |  |  | 0.09 (−0.25–0.43)^e^ |  | Dif score: 0.02, s.d. of DV: 0.23^f^ |

Significant effect sizes are marked with bold.

V: video; Q: questionnaire.

DV, dependent variable; DMC, Dyadic Mutuality Code; NCATS, Nursing Child Assessment Teaching Scale; NCAST PCI, Nursing child Assessment Satellite Training; EAS, Emotional Availability Scales; CIB, Coding Interactive Behavior; ASCT, Attachment Story Completion Task; AQS, Attachment Q-set; ASSP, Ainsworth Strange Situation Procedure.

a. Pooled effect size of all outcomes entered in meta-analysis.

b. Only Global rating scale Murray Mother–infant relationship was entered in the meta-analysis.

c. Adjusted for relationship problems prior to treatment.

d. Adjus ted for maternal baseline depressive symptoms.

e. Active control group – two interventions are compared.

f. Adjusted for infant age and gender, infant temperament, postnatal depression severity and socioeconomic status.

Table 3 Child development outcomes as reported across studies in the systematic review

| Study | Measure | Assessment | Child age in months | Intervention | | | Control | | | Effect size | | Other statistics |
| --- | --- | --- | --- | --- | --- | --- | --- | --- | --- | --- | --- | --- |
|  |  |  |  | *N* | Mean | s.d. | *N* | Mean | s.d. | Cohen’s d (95% CI) | RR (95% CI) |  |
| Post-intervention | | | | | | | | | | | | |
| Letournau 2011 | MDI | O | ~8 | 21 | 97.4 | 12.2 | 27 | 100.2 | 13.6 | −0.22 (−0.79; 0.36) |  |  |
|  | ICQ Socioemotional development^a^ | Q | ~8 | 23 | 72 | 20.6 | 28 | 65.6 | 18.2 | −0.33 (−0.89; 0.22) |  |  |
| Murray 2003 | Behaviour problems (in-house) | Q | 4.5 |  |  |  |  |  |  |  |  |  |
| Counselling |  |  |  | 40 |  |  | 31 |  |  |  | 0.91 (0.42–1.58)^b^ |  |
| Psyhodynamic |  |  |  | 40 |  |  | 31 |  |  |  | 1.21 (0.621.87)^b^ |  |
| Cognitive behavioural |  |  |  | 41 |  |  | 31 |  |  |  | 0.83 (0.37–1.50)^b^ |  |
| Short term | | | | | | | | | | | | |
| Van Doesum 2008 | ITSEA Externalising^a^ | Q | ~18 |  | 0.60 | 0.39 |  | 0.57 | 0.30 | −0.09 (−0.55; 0.38) |  |  |
|  | ITSEA Internalizing^a^ | Q | ~18 |  | 0.45 | 0.23 |  | 0.39 | 0.16 | −0.30 (−0.77; 0.16) |  |  |
|  | ITSEA Dysregulation^a^ | Q | ~18 |  | 0.46 | 0.36 |  | 0.48 | 0.26 | 0.06 (−0.40; 0.53) |  |  |
|  | ITSEA Competence | Q | ~18 |  | 1.40 | 0.28 |  | 1.22 | 0.30 | **0.62 (0.14; 1.10)** |  |  |
| Long term | | | | | | | | | | | | |
| Kersten-Alvarez 2010 | Self-esteem (puppet interview) | O | ~68 | 29 |  |  | 29 |  |  | 0.29 (−0.23; 0.80) |  | Dif score:0.43; s.d. of DV:1.53^c^ |
|  | California Child Q-Set ego-resiliency | Q | ~68 | 28 |  |  | 29 |  |  | 0.29 (−0.24; 0.81) |  | Dif score: 0.09, s.d. of DV: 0.32^c^ |
|  | PPVT-R Verbal intelligence | T | ~68 | 29 |  |  | 29 |  |  | 0.06 (−0.45; 0.58) |  | Dif score: 1.16, s.d. of DV: 18.89^c^ |
|  | PSBQ prosocial | Q | ~68 | 28 |  |  | 29 |  |  | −0.11 (−0.63; 0.41) |  | Dif score: −0.67, s.d. of DV: 6.13^c^ |
|  | SRS School Adjustment | Q | ~68 | 28 |  |  | 29 |  |  | 0.08 (−0.44; 0.59) |  | Dif score:0.83, s.d. of DV:11.18^c^ |
|  | CBCL Internalizing^a^ | Q | ~68 | 28 |  |  | 29 |  |  | −0.35 (−0.87; 0.17) |  | Dif score: 3.94, s.d. of DV: 11.56 |
|  | CBCL Externalizing^a^ | Q | ~68 | 28 |  |  | 29 |  |  | 0.02 (−0.50; 0.54) |  | Dif score: −0.24, s.d. of DV: 11.28 |
|  | C-TRF Internalizing^a^ | Q^d^ | ~68 | 28 |  |  | 29 |  |  | −0.16 (−0.68; 0.36) |  | Dif score: 1.40, s.d. of DV: 9.07^c^ |
|  | C-TRF Externalizing | Q^d^ | ~68 | 28 |  |  | 29 |  |  | −0.23 (−0.76; 0.29) |  | Dif score: 2.06, s.d. of DV: 8.93^c^ |
| Murray 2003 | BSQ Emotional and behavioural problems^a^ | Q | 18 |  |  |  |  |  |  |  |  |  |
| Counselling |  |  |  | 46 | 4.28 | 2.6 | 48 | 6.21 | 3.40 | **0.64 (0.22; 1.05)** |  |  |
| Psyhodynamic |  |  |  | 42 | 4.62 | 3.09 | 48 | 6.21 | 3.40 | **0.49 (0.07; 0.91)** |  |  |
| Cognitive behavioural |  |  |  | 42 | 5.19 | 3.07 | 48 | 6.21 | 3.40 | 0.31 (−0.10; 0.73) |  |  |
|  | MDI | O | 18 |  |  |  |  |  |  |  |  |  |
| Counselling |  |  |  | 46 | 111.96 | 21.07 | 48 | 115.29 | 16.02 | −0.18 (−0.58; 0.23) |  |  |
| Psyhodynamic |  |  |  | 42 | 115.33 | 17.21 | 48 | 115.29 | 16.02 | 0.00 (−0.41; 0.42) |  |  |
| Cognitive behavioural |  |  |  | 42 | 115.17 | 17.71 | 48 | 115.29 | 16.02 | −0.00 (−0.42; 0.41) |  |  |
|  | Rutter A^2^ Emotional and behavioural difficulties^a^ | Q | 60 |  |  |  |  |  |  |  |  |  |
| Counselling |  |  |  | 33 | 11.12 | 6.77 | 35 | 11.19 | 5.17 | 0.01 (−0.46; 0.49) |  |  |
| Psyhodynamic |  |  |  | 28 | 11.79 | 6.23 | 35 | 11.19 | 5.17 | −0.11 (−0.60; 0.39) |  |  |
| Cognitive behavioural |  |  |  | 31 | 8.29 | 4.16 | 35 | 11.19 | 5.17 | **0.61 (0.12; 1.11)** |  |  |
|  | PBCL | Q^d^ | 60 |  |  |  |  |  |  |  |  |  |
| Counselling |  |  |  | 26 | 4.38 | 3.60 | 33 | 5.67 | 6.38 | 0.24 (−0.27; 0.76) |  |  |
| Psyhodynamic |  |  |  | 28 | 4.46 | 3.06 | 33 | 5.67 | 6.38 | 0.24 (−0.27; 0.74) |  |  |
| Cognitive behavioural |  |  |  | 29 | 4.45 | 3.60 | 33 | 5.67 | 6.38 | 0.23 (−0.27; 0.73) |  |  |
|  | McCarthy, GCI |  | 60 |  |  |  |  |  |  |  |  |  |
| Counselling |  |  |  | 33 | 106.67 | 20.91 | 39 | 107.92 | 16.90 | −0.07 (−0.53; 0.40) |  |  |
| Psyhodynamic |  |  |  | 32 | 106.78 | 15.21 | 39 | 107.92 | 16.90 | −0.07 (−0.54; 0.40) |  |  |
| Cognitive behavioural |  |  |  | 35 | 110.17 | 17.56 | 39 | 107.92 | 16.90 | 0.13 (−0.33; 0.59) |  |  |
| Stein 2018 | BSID-III Cognitive | O | ~24 | 62 |  |  | 67 |  |  | −0.08 (−0.43–0.26)^e^ |  | Dif score: −1.01, s.d. of DV:12.46^f^ |
|  | BSID-III Language | O | ~24 | 62 |  |  | 67 |  |  | 0.11 (−0.23–0.46)^e^ |  | Dif score: 1.33, s.d. of DV: 12.01^f^ |
|  | CBCL externalizing^a^ | Q | ~24 | 64 |  |  | 68 |  |  | 0.22 (−0.12–0.57)^e^ |  | Dif score: −1.77, s.d. of DV: 8.0^f^ |
|  | ECBQ Attention focusing | Q | ~24 | 63 |  |  | 68 |  |  | 0.05 (−0.30–0.40)^e^ |  | Dif score: 0.04, s.d. of DV: 0.75^f^ |
|  | ECBQ Attentional shifting | Q | ~24 | 63 |  |  | 68 |  |  | 0.27 (−0.07–0.61)^e^ |  | Dif score: 0.16, s.d. of DV: 0.60^f^ |
|  | ECBQ Inhibitory control | Q | ~24 | 63 |  |  | 68 |  |  | 0.08 (−0.26–0.42)^e^ |  | Dif score: 0.07, s.d. of DV: 0.90^f^ |
|  | Lab-TAB emotion regulation^a^ | O | ~24 | 61 |  |  | 62 |  |  | 0.31 (−0.04–0.67)^e^ |  | Dif score: −0.32, s.d. of DV: 1.04^f^ |

Significant effect sizes are marked with bold.

O: Observation; Q: questionnaire; T: test, V: video.

DV, dependent variable; MDI, Mental Development Index (BSID); ICQ, Infant Characteristics Questionnaire; ITSEA, Infant Todler Social and Emotional Assessment; PPVT-R, Peabody Picture Vocabulary Test – Revised; PSBQ, Preschool Social Behavior Questionnaire; SRS, Stress Response Scale; CBCL, Child Behavior Checklist; C-TRF, Caregiver-Teacher Report Form; BSQ, Behavioural Screening Questionnaire; PBCL, Pre-school Behaviour Checklist; GCI, General Cognitive Index; BSID-III, Bayley Scales of Infant Development; ECBQ, Early Childhood Behavior Questionnaire; Lab-TAB, Laboratory Temperament Assessment Battery.

a. Reverse scoring – high score is negative.

b. Adjusted for behavioural management problems prior to treatments.

c. Adjusted for maternal baseline depressive symptoms.

d. Teacher reported.

e. Active control group – two interventions are compared.

f. Adjusted for infant age and gender, infant temperament, postnatal depression severity and socioeconomic status.
