# Supplementary material for: Effects of parenting interventions for mothers with depressive symptoms and an infant: systematic review and meta-analysis
Source: BJPsych Open. 2020 Jan 13;6(1):e9. doi: 10.1192/bjo.2019.89 (PMC7001473; doi:10.1192/bjo.2019.89)
Supplement: Supplementary file 1 [file S2056472419000899sup001.zip › BJO_1900089_supplementaryTable1.docx]

**Supplementary Table 1 Risk of bias of included studies for child development and parent-child relationship outcomes**

|  | **Outcome measure/Risk of bias domains** | Sequence generation | Allocation concealment | Blinding of outcome assessor | Incomplete outcome data addressed | Free of selective reporting | A priori protocol | Free of other bias |
| --- | --- | --- | --- | --- | --- | --- | --- | --- |
| **Child development** |  |  |  |  |  |  |  |  |
| Kersten-Alvarez et al. 2010* | Puppet interview (self-esteem), Child Q-set (ego-resiliency), PPVT-R - subscale of PSBQ (verbal intelligence), Subscale of PSBQ (prosocial behavior), Subscale of Stress response scale (school adjustment), CBCL (behavior problems) | L | L | 1 | 4 | U | Yes | 1 |
| Letourneau et al. 2011 |  | U | L | - | - | - | U | - |
|  | BSID/MDI (cognitive development) | - | - | 1 | 2 | U | - | 2 |
|  | ICQ (socioemotional development) | - | - | 3 | 1 |  | - | 3 |
| van Doesum et al. 2008* | ITSEA/externalizing/internalizing/dysregulation/competence) | L | L | 1 | 2 | U | U | 1 |
| Murray et al. 2003 |  | L | U | - | - | - | U | - |
|  | Homemade checklist (behavior management problems) | - | - | 3 | 1 | U | - | 1 |
|  | BSQ (emotional and behavioral problems) | - | - | 3 | 1 | U | - | 1 |
|  | BSID/MDI (cognitive development) | - | - | 1 | 1 | 3 | - | 1 |
|  | Rutter scale A2 (emotional and behavioral difficulties) | - | - | 3 | U | U | - | 1 |
|  | PBCL (emotional and behavioral difficulties) | - | - | 1 | U | 3 | - | 1 |
|  | GCI, McCarthy (cognitive development) | - | - | 1 | U | 3 | - | 1 |
| Stein et al. 2018 |  | L | L | - | - | - | Yes | - |
|  | BSID-III ( Child cognitive and language development) | - | - | 1 | 2 | 1 | - | 2 |
|  | CBCL/Externalizing | - | - | 3 | 2 | 1 | - | 2 |
|  | ECBQ (Attention focusing, attention shifting, Inhibitory control | - | - | 3 | 2 | 1 | - | 2 |
|  | Lab-TAB (Child emotion regulation) | - | - | 1 | 2 | 1 | - | 2 |
| **Parent-child relationship** |  |  |  |  |  |  |  |  |
| Horowitz et al. 2001 | DMC (responsiveness) | L | L | 1 | 2 | U | U | 1 |
| Horowitz et al. 2013 | NCATS (relational effectiveness, child responsiveness) | U | U | 1 | 3 | U | U | 3 |
| Kersten-Alvarez et al. 2010* | Two scales: 1.Erickson and 2. Smeekens (maternal interactive behavior), Adapted version of ASCT (attachment security) | L | L | 1 | 4 | U | Yes | 1 |
| Letourneau et al. 2011 |  | U | L | - | - | - | U | - |
|  | NCAST, PCI feeding (mother-infant interaction) | - | - | 1 | 3 | U | - | 2 |
|  | NCAST, PCI teaching (mother-infant interaction) | - | - | 1 | 2 | U | - | 2 |
| van Doesum et al. 2008* | EAS (maternal sensitivity, maternal structuring, maternal non-intrusiveness, maternal non-hostility, child responsiveness, child involvement), AQS (child attachment security) | L | L | 1 | 2 | U | U | 1 |
| Murray et al. 2003 |  | L | U | - | - | - | U | - |
|  | Homemade checklist (parent-infant relationship problems) | - | - | 3 | 1 | U | - | 1 |
|  | Global rating scale by Murray (mother-infant relationship) | - | - | 1 | 1 | U | - | 1 |
|  | ASSP (infant attachment) | - |  | 1 | 1 | U | - | 1 |
| Goodman et al. 2015* | CIB (maternal sensitivity, infant involvement, dyadic reciprocity) | L | L | 1 | 1 | 3 | U | 3 |
| Stein et al. 2018 | Attachment Q Sort (attachment security) | L | L | 1 | 2 | 1 | Yes | 2 |

*Note: Risk of bias was conducted for each outcome. When risk of bias was the same for all included outcomes, only one score is provided in the table.

Note: In the 5-point scale 1 corresponds to low risk of bias and 5 correspond to high risk of bias. L= low risk of bias; H=high risk of bias; U= unclear risk of bias

DMC: The dyadic mutuality code, NCATS: The nursing child assessment teaching scale, PCI: Parent-Child Interaction - Feeding and Teaching Scales, ASCT: Attachment Story Completion Task, PPVT-R: The Peabody Picture Vocabulary Test, revised edition, PSBQ: Preschool Social Behavior Questionnaire, CBCL: Child Behavior Checklist, MDI: Bayley score on the Mental Development Index, ICQ: Infant Characteristics Questionnaire, ITSEA: The Infant Toddler Social Emotional Assessment, EAS: Emotional Availability Scales, AQS: Attachment Q Sort, BSQ: Behavioral Screening Questionnaire, PBCL: Preschool Behavior Checklist, CGI: General Cognitive Index, ASSP: Ainsworth Strange Situation procedure, CIB: the Coding Interactive Behavior scale
