# Supplementary material for: Effects of parenting interventions for mothers with depressive symptoms and an infant: systematic review and meta-analysis
Source: BJPsych Open. 2020 Jan 13;6(1):e9. doi: 10.1192/bjo.2019.89 (PMC7001473; doi:10.1192/bjo.2019.89)
Supplement: Supplementary file 1 [file S2056472419000899sup001.zip › For Proof_Supplementary file 1 Pubmed search.pdf]

Supplementary file 1 Electronic search strategy for Pubmed search

((((((((((((((primipara[Title/Abstract]) OR ("low birth weight" OR LBW[Title/Abstract])) OR (baby OR babies[Title/Abstract])) OR "born prematurely"[Title/Abstract]) OR father*[Title/Abstract]) OR infancy[Title/Abstract]) OR (infant OR infants[Title/Abstract])) OR mother*[Title/Abstract]) OR (neonate OR neonates OR neonatal OR neonatal[Title/Abstract])) OR newborn*[Title/Abstract]) OR perinatal[Title/Abstract]) OR toddler*[Title/Abstract]) OR (((preterm[Title/Abstract]) AND (infant OR infants[Title/Abstract])) OR (((teenage* OR adolescent*[Title/Abstract])) AND (pregnancy OR pregnancies[Title/Abstract])) OR (((postnatal OR "post natal" OR "post-natal" OR maternal[Title/Abstract])) AND depression[Title/Abstract])))) OR (((((((("Mothers"[Mesh]) OR "Pregnancy in Adolescence"[Mesh]) OR "Infant, Low Birth Weight"[Mesh]) OR "Fathers"[Mesh]) OR "Depression, Postpartum"[Mesh]) OR "Premature Birth"[Mesh]) OR "Parents"[Mesh]) OR "Single Parent"[Mesh])

AND

((((((((((((((((((((((("Attachment Behavior"[Title/Abstract]) OR "Behavior Disorders"[Title/Abstract]) OR ("Behavior Problems" OR "Behaviour Problems"[Title/Abstract])) OR "Early Childhood Development"[Title/Abstract]) OR "Emotional Adjustment"[Title/Abstract]) OR (externalization OR externalization[Title/Abstract])) OR "Parental Attitudes"[Title/Abstract]) OR "Parenting Skills"[Title/Abstract]) OR "Psychosocial Development"[Title/Abstract]) OR "Self Confidence"[Title/Abstract]) OR ("family relation" OR "family relations" OR "family relationship"[Title/Abstract])) OR "infant mental health"[Title/Abstract]) OR "Parent relationship"[Title/Abstract]) OR "sense of coherence"[Title/Abstract]) OR (attach OR attaches OR attached OR attachment[Title/Abstract])) OR "parent practice"[Title/Abstract]) OR soc13[Title/Abstract]) OR kpcs[Title/Abstract]) OR "karitane parenting confidence scale"[Title/Abstract]) OR "bayley scales"[Title/Abstract]) OR "The Ages & Stages Questionnaire"[Title/Abstract]) OR asq[Title/Abstract]) OR (itsea OR bitsea[Title/Abstract]))) OR (((((((("Self Concept"[Mesh]) OR "Mother-Child Relations"[Mesh]) OR "Father-Child Relations"[Mesh]) OR "Parent-Child Relations"[Mesh]) OR "Child Development"[Mesh]) OR "Conduct Disorder"[Mesh]) OR "Child Behavior Disorders"[Mesh]) OR "Parenting"[Mesh])

AND

((((((((((((((((((((((((((((("emotional and behavioral adjustment" OR "emotional and behavioural adjustment"[Title/Abstract])) OR ("Child Psychotherapy" OR "play therapy"[Title/Abstract])) OR "early intervention"[Title/Abstract]) OR "family intervention"[Title/Abstract]) OR "Family Systems Theory"[Title/Abstract]) OR "Conjoint Therapy"[Title/Abstract]) OR "Strategic Family Therapy"[Title/Abstract]) OR "Structural Family Therapy"[Title/Abstract]) OR "Childbirth Training"[Title/Abstract]) OR "Skill Learning"[Title/Abstract]) OR "Social Skills Training"[Title/Abstract]) OR ("home visit" OR "home visits"[Title/Abstract])) OR "manual based"[Title/Abstract]) OR "parent education"[Title/Abstract]) OR ("pediatric intervention" OR "pediatric interventions"[Title/Abstract])) OR "primary intervention"[Title/Abstract]) OR ("supporting parent" OR "supporting parents"[Title/Abstract])) OR "parent intervention"[Title/Abstract]) OR ("behavior modification" OR "behaviour modification"[Title/Abstract])) OR "home based intervention"[Title/Abstract]) OR parenting[Title/Abstract]) OR preventing[Title/Abstract]) OR videotap*[Title/Abstract]) OR (psychotherapy OR psychotherapies[Title/Abstract])) OR (therapy OR therapies[Title/Abstract]))) OR ((((family OR parenting[Title/Abstract])) AND (program OR programme OR programmes OR programs[Title/Abstract])) OR (((father OR fathers OR mother OR mothers OR parent OR parents OR parental[Title/Abstract])) AND training[Title/Abstract]) OR (((maternal OR paternal OR parental[Title/Abstract])) AND learning[Title/Abstract]) OR (((feedback OR course[Title/Abstract])) AND video[Title/Abstract]) OR ((((mother OR father OR parent OR parental[Title/Abstract])) AND infant[Title/Abstract]) AND interaction[Title/Abstract]) OR (((nurse[Title/Abstract]) AND family[Title/Abstract]) AND partnership[Title/Abstract]) OR ((nurse[Title/Abstract]) AND visit*[Title/Abstract]))) OR ((((("Behavior Therapy"[Mesh]) OR "Psychotherapy, Brief"[Mesh]) OR "Family Therapy"[Mesh]) OR "Psychotherapy, Group"[Mesh]) OR "Prenatal Care"[Mesh])

AND

((((((((((“randomized controlled trial” or “Experimental Design” or “Between Groups De-sign” or “Experiment Controls” or “Quasi Experimental Methods” or “Experimental Methods”[Title/Abstract]))))) OR (((quasi-experiment* or quasiexperiment* or (propensity score*) or (compar* AND group*) or (match* AND control*) or (match* AND group*) or (match* AND compar*) or (clinical AND trial*) or (experiment* AND trial*) or

(experiment* AND design*) or (experiment* AND method*) or (experiment* AND stud*) or (experiment* AND evaluation*) or (experiment* AND test*) or (experiment* AND assessment*) or “assessment only” or “comparison sample” or propensity-matched or (between AND group*) or longi-tud*)[Title/Abstract]))) OR (((Non-random* or nonradom* or (non AND random*))[Title/Abstract]))) OR ((((control or treatment or experiment* or intervention or assign*) AND (group* or subject* or patient* or intervention))[Title/Abstract]))) OR rct) OR ”Random Allocation”[Title/Abstract]) OR ((randomized controlled trial[MeSH Terms]) OR propensity score[MeSH Terms]) OR (((systematic OR literature[Title/Abstract])) AND review*[Title/Abstract])
